# Obesity Is Not Associated with Impaired Immune Response to Influenza Vaccination in HIV-Infected Persons

**DOI:** 10.1155/2015/653840

**Published:** 2015-10-12

**Authors:** Charitha Gowda, Noah McKittrick, Deborah Kim, Rosemarie A. Kappes, Vincent Lo Re, Pablo Tebas

**Affiliations:** ^1^Division of Infectious Diseases, Department of Medicine, Perelman School of Medicine, University of Pennsylvania, Philadelphia, PA 19104, USA; ^2^Department of Biostatistics and Epidemiology and Center for Clinical Epidemiology and Biostatistics, Perelman School of Medicine, University of Pennsylvania, Philadelphia, PA 19104, USA; ^3^Division of Infectious Diseases, Stanford University School of Medicine, Stanford, CA 94305, USA

## Abstract

*Introduction*. HIV-infected individuals demonstrate lower immunogenicity to the influenza vaccine, despite immunologic and virologic control of HIV infection. Obesity has been previously shown to be associated with diminished antibody responses to other vaccines in HIV-uninfected persons. However, no studies have examined if obesity is associated with diminished protective immune response to influenza vaccination among HIV-infected persons on antiretroviral therapy (ART). *Methods*. We performed a retrospective analysis of immunogenicity data from a clinical trial of inactivated, trivalent influenza vaccine. The primary endpoint was the proportion of participants with seroconversion, defined as >4-fold increase in anti-hemagglutinin antibody titers after vaccination. Secondary endpoints were the proportion of participants with seroprotection (defined as antibody titers of ≥1 : 40) and geometric mean hemagglutination inhibition antibody titers. *Results*. Overall, 48 (27%) participants were obese (body mass index ≥ 30 kg/m^2^). Seroconversion rates were comparable between obese and nonobese subjects for all three vaccine strains. Further, postvaccination geometric mean titers did not differ by body mass index category. *Conclusion*. Obesity was not associated with diminished antibody response to influenza vaccination in a sample of healthy HIV-infected persons.

## 1. Introduction

Influenza is responsible for substantial morbidity and mortality worldwide, causing over 250,000 deaths annually [1]. HIV-infected individuals are particularly vulnerable to serious complications of influenza [2, 3], but they have lower serological responses to the influenza vaccine compared to the general population [4–6]. While low CD4 cell counts and HIV viremia are important determinants of vaccine response, they do not fully explain the reduced immunogenicity to influenza vaccine observed in this population, indicating that other factors contribute as well [5, 6].

Obesity may be one such factor affecting vaccine immunogenicity, as it has been previously implicated in reduced immunogenicity to the hepatitis B vaccine in healthy, uninfected adults and to tetanus toxoid in healthy adolescents [7–10]. In addition, reduced immune responses to a candidate HIV-1 vaccine were seen among overweight, uninfected adults [11]. Obesity is associated with a chronic inflammatory state that could lead to impairments in innate and adaptive immune function [10, 12–14]. Further, excess peripheral adiposity may lead to inadequate vaccine delivery intramuscularly, reducing antigen uptake and presentation to the immune system [15].

The potential impact of obesity on influenza vaccine immunogenicity is of particular interest since influenza infection has been shown to be more severe among overweight and obese individuals [14, 16–19]. However, prior studies of influenza vaccine in HIV-uninfected individuals have demonstrated conflicting data regarding the impact of obesity on vaccine immunogenicity [20–23]. Two studies among elderly patients found no association between body mass index (BMI) and influenza vaccine immunogenicity [21, 23]. In contrast, another study of healthy adults ≥18 years old observed greater declines in antibody titers over time with increasing BMI, despite higher influenza antibody responses immediately after vaccination [22]. Further, pooled data from 3 trials of H1N1 influenza vaccine immunogenicity showed that one vaccine dose was associated with higher antibody titers in obese compared to nonobese adults, but subsequently there were no significant differences in vaccine responsiveness by BMI following the two doses [20]. To our knowledge, no studies to date have explored the relationship between obesity and influenza vaccine response specifically among HIV-infected individuals.

With the advent of ART and the fact that HIV infection disproportionately affects underserved populations with limited access to healthy diets, obesity has become more prevalent among HIV-infected individuals [24–28]. Given that HIV-infected individuals are already more likely to have impaired vaccine responses due to their underlying immunocompromised state, identifying potentially reversible factors, such as obesity, that may contribute to weakened vaccine immunogenicity is critically important. This study evaluated if obesity was associated with diminished vaccine responsiveness to the 2010-2011 seasonal influenza vaccine in HIV-infected persons and examined whether a high dose vaccine formulation could improve immunogenicity among obese individuals.

## 2. Methods

We performed a retrospective analysis of a previously reported double-blinded, randomized, controlled trial of standard dose (SD) versus high dose (HD) influenza vaccine in HIV-infected persons [29]. From October 27, 2010, to March 27, 2011, the trial enrolled HIV-infected adults (18 years or older) seen at the MacGregor Clinic of the Hospital of the University of Pennsylvania in Philadelphia, PA. The study was approved by the University of Pennsylvania Institutional Review Board.

Participants were randomized to receive either the SD formulation of the licensed, inactivated, trivalent, unadjuvanted influenza vaccine (Fluzone, lot U3774DA) or the HD formulation (Fluzone High Dose, lot U3635AA), which contained 15 mcg and 60 mcg, respectively, of hemagglutinin from the following three strains: A/California/07/2009 X-179A (H1N1), A/Victoria/210/2009 X-187 (H3N2), and B/Brisbane/60/2008 (influenza B). Vaccine was administered intramuscularly in the deltoid muscle using standard-length (5/8- or 1-inch) needles, and participants provided serum samples prior to and 21 to 28 days after vaccination. Hemagglutination inhibition (HAI) assays against the 3 vaccine components (H1N1, H3N2, and influenza B) were performed, as described previously [29], on the pre- and postvaccination serum samples.

The primary immunogenicity endpoint was the proportion of participants with seroconversion after vaccination. Seroconversion was defined as a 4-fold rise in hemagglutination inhibition (HAI) antibody titer if the baseline titer was ≥1 : 10 or a postvaccination HAI titer ≥1 : 40 if the baseline titer <1 : 10. Secondary endpoints were the proportion of participants with seroprotection after immunization, defined as a postvaccination HAI antibody titer ≥1 : 40, as well as pre- and postvaccination geometric mean titers (GMT) of HAI antibody.

Height and weight measurements were obtained from an examination performed at enrollment or during the nearest clinic visit within 3 months. BMI was calculated as weight (kg)/height (m)^2^, with obesity defined by a BMI ≥ 30 kg/m^2^. Demographic and clinical variables collected included age, race/ethnicity, ART use, presence of diabetes, hepatitis B or C coinfections, insulin therapy, current and nadir CD4 cell counts, and HIV viral load.

Differences in characteristics between nonobese and obese participants were assessed using Chi-square tests for categorical data and Wilcoxon rank-sum tests for continuous data. Proportions and 95% confidence intervals (CIs) of participants with seroconversion and seroprotection were calculated separately and between-group differences evaluated by Chi-square test. It was predetermined that a sample of 192 participants would provide 80% power to determine a 20% difference in seroconversion rates, assuming a 25% prevalence of obesity and two-tailed alpha of 0.05. To exclude the possibility that obesity was a marker of preserved or reconstituted immune function, analyses were repeated among participants with CD4 cell counts > 200 cells/*μ*L and >500 cells/*μ*L. Log transformation was performed to normalize the distribution of HAI antibody titers, and Student's* t*-test was used to compare GMTs between nonobese and obese groups for each vaccine strain. Although multiple comparisons were performed across correlated immunogenicity measurements, Bonferroni correction was not appropriate because it assumes that these measurements are independent. We interpreted findings with caution and examined the consistency of results across vaccine components.

## 3. Results

Of the 195 HIV-infected persons studied in the original clinical trial, data on BMI and influenza vaccine response were available for 176 persons (85 in the SD group; 91 in the HD group). The characteristics of the study sample are reported in Table 1. Overall, 48 (27%) participants were obese. There were significantly more males (*p* < 0.001) and fewer diabetics (*p* = 0.002) in the nonobese group. While the prevalence of chronic hepatitis C infection did not differ, chronic hepatitis B was more prevalent among nonobese than obese participants (7.8% versus 0%; *p* = 0.046).

The majority were on a protease inhibitor (PI) or nonnucleoside reverse transcriptase inhibitor- (NNRTI-) based ART regimen, and the distribution of ART regimens was similar between the obese and nonobese groups (*p* = 0.56; Table 1). There were no differences between the groups with respect to median nadir CD4 (*p* = 0.25) and current CD4 (*p* = 0.09) counts or the proportion of participants with HIV RNA < 400 copies/mL (*p* = 0.66; Table 1).

Seroconversion and seroprotection rates are presented in Table 2. There were no significant differences in the proportions of participants who seroconverted between the obese and nonobese groups for all three vaccine strains: H1N1 (62% [48–77] versus 70% [62–78]; *p* = 0.32), H3N2 (79% [67–91] versus 77% [69–84]; *p* = 0.71), and influenza B (44% [35–52] versus 48% [33–63]; *p* = 0.62). An equal proportion of nonobese and obese participants received the HD formulation (50% versus 56%; *p* = 0.46; Table 1). In both groups, seroconversion rates were higher after HD compared to SD vaccination (Table 2).

Proportions of participants with seroprotection after vaccination ranged from 67 to 100% depending on the influenza strain or vaccine formulation (Table 2). Seroprotection rates did not differ by BMI for those who received the SD vaccine. A lower proportion of obese individuals who received the HD vaccine had seroprotection after vaccination against the H1N1 strain only (89% [95% CI, 76 to 100%] versus 98% [95% CI, 95 to 100%]; *p* = 0.04). When these analyses were repeated among those with CD4 counts > 200 cells/*μ*L (*n* = 157) and CD4 counts > 500 cells/*μ*L (*n* = 78), seroconversion and seroprotection rates did not differ by obesity for all three influenza strains (Table 3).

Pre- and postvaccination GMTs are reported in Table 2. For all 3 vaccine components, there were no statistically significant differences in GMTs between obese versus nonobese participants who received the SD vaccine. Among those who received the HD vaccine, obese participants had a higher baseline GMT against the H1N1 strain compared to normal-weight persons (baseline GMT, 17 [9–30] versus 59 [23–154]; *p* = 0.02). However, postvaccination GMTs did not differ between nonobese and obese participants for all 3 vaccine components.

## 4. Discussion

To our knowledge, this is the first study to evaluate if obesity influences influenza vaccine immunogenicity in HIV-infected individuals. Our results showed that obese, HIV-infected persons did not have impaired serologic responses to influenza vaccination when compared to nonobese HIV-infected individuals.

Our findings on influenza vaccine immunogenicity in obese, HIV-infected participants are consistent with the results from similar investigations in obese, HIV-uninfected persons. Two studies of older, HIV-uninfected adults found no association between obesity and influenza vaccine seroprotection immediately after vaccination [21, 23]. In another study of healthy adults without chronic disease, higher HAI antibody titers to influenza vaccine were found one month after vaccination in obese individuals compared to normal-weight adults, although a greater decline in antibody titers was seen after 12 months in the obese group [22]. Thus, despite exhibiting impaired immune function following influenza infection [14, 16–19], obese individuals may not demonstrate reduced vaccine immunogenicity after vaccination, independent of the presence of HIV infection. However, it remains unclear if influenza vaccine protection wanes more over time in obese individuals, and this should be evaluated in future studies.

Further investigation is warranted to evaluate why obesity does not appear to impair immune response to the influenza vaccine in both HIV-infected and -uninfected persons. There are several potential reasons why vaccine immunogenicity may be unaffected by BMI among HIV-infected individuals. Immune impairment from obesity may be negligible compared to the underlying immune dysfunction caused by HIV infection or inadequate immune recovery seen with ART [30]. Second, HIV infection independently leads to chronic inflammation [31], and obesity may not contribute additionally to immune dysfunction through this mechanism. Finally, ART use has been associated with peripheral lipoatrophy, which may minimize the likelihood of inadequate vaccine delivery in obese, HIV-infected individuals [32]. In this study, there were no differences between obese and nonobese participants in the distribution of ART regimens used or the proportion taking zidovudine, didanosine, or stavudine, drugs that have been previously implicated in lipoatrophy [33, 34], suggesting that the type of ART may not have contributed significantly to body composition changes. However, body composition of participants was not formally evaluated, precluding us from making any conclusions on the impact of altered body fat distribution on vaccine immunogenicity in this population.

With HIV infection, a paradoxical effect may be expected where obesity is associated with improved vaccine response. Since uncontrolled HIV infection often causes substantial weight loss, obesity could have been a surrogate marker for immunologic control, leading obese individuals to have increased vaccine immunogenicity compared to their normal-weight counterparts. However, even after restricting analyses to participants with preserved immune function (CD4 > 200 cells/*μ*L and >500 cells/*μ*L), there were no significant differences by BMI in serologic responses after vaccination. Thus, our study adds to the growing body of literature that suggests that obesity is not a major factor influencing host response to influenza vaccination, independent of HIV status. Compared to other vaccines, there may be innate differences to the influenza vaccine, with respect to vaccine composition, production, or administration that account for the overall lack of association between obesity and vaccine immunogenicity and should be evaluated within future studies.

Of those who received the HD vaccine, obese participants had a statistically significantly lower seroprotection rate for the H1N1 strain, but not the H3N2 or influenza B strain, than nonobese participants. Given the increased morbidity and mortality to the 2009 pandemic H1N1 influenza infection observed in obese persons due to, in part, the impaired immune response [19, 35], it is possible that immunogenicity to the H1N1 vaccine component could be similarly attenuated by obesity. Obese participants had higher prevaccination but not postvaccination GMT titers against H1N1, which may help explain why there was no observed association between obesity and the seroconversion rate for the H1N1 strain. However, it is unclear why such an association would not be present in those who received the SD vaccine. Follow-up studies are needed to determine if indeed the H1N1 influenza strain differentially affects obese individuals or this simply represents a chance finding.

Our investigation has several potential limitations. Height and weight measurements used to determine BMI were collected within a 3-month period around vaccination date. However, 74% of participants had anthropometric measurements collected within one week of vaccination. Additionally, since the majority of participants were maintained on stable ART, clinically meaningful changes in weight during such a short time period were not expected [36, 37]. As with other influenza vaccine trials, vaccine effectiveness was ascertained using serologic responses as surrogate measures, which was more feasible than measuring incidence of influenza cases. Further, our study was not powered to detect less than 20% difference in seroconversion rates between obese and nonobese participants. Lastly, since the majority of study participants were men, whose body composition may differ substantially from women, the study's findings may not be generalizable to women.

In summary, obesity was not independently associated with serologic response to any of the antigens in the 2010-2011 inactivated, trivalent influenza vaccine in a predominantly male sample of well-controlled HIV-infected individuals. This suggests that the increasing prevalence of obesity in the HIV-infected population may not necessarily mandate changes to influenza vaccination strategies, such as more frequent dosing or larger antigen doses, in order to improve vaccine efficacy.

## Figures and Tables

**Table 1:** Baseline characteristics of the study sample, overall and by obese status.

	Overall (*n* = 176)	Nonobese (BMI < 30 kg/m^2^) (*n* = 128)	Obese (BMI ≥ 30 kg/m^2^) (*n* = 48)	*p* value
Median age (IQR), year	46 (38–52)	46 (37–53)	44 (38–51)	0.56
Sex, *n* (%)				
Male	125 (71)	102 (80)	23 (48)	<0.001
Female	51 (29)	26 (20)	25 (52)	
Race, *n* (%)				
White	54 (31)	44 (34)	10 (21)	0.17
Black	121 (68)	83 (65)	38 (79)	
Asian/Pacific-Islander	1 (0.6)	1 (0.8)	0 (0)	
Diabetes, *n* (%)	12 (6.8)	4 (3.1)	8 (16.7)	0.002
Chronic hepatitis B, *n* (%)	10 (5.7)	10 (7.8)	0 (0)	0.05
Chronic hepatitis C, *n* (%)	25 (14)	19 (15)	6 (12)	0.69
Receiving ART, *n* (%)	156 (87)	114 (89)	42 (88)	0.77
PI-based	85 (48)	63 (49)	22 (46)	0.56
NNRTI-based	43 (24)	30 (23)	13 (27)	
INSTI-based	10 (5.7)	8 (6.2)	2 (4.2)	
Other	24 (14)	15 (12)	9 (19)	
No ART/unknown	14 (8.0)	12 (9)	2 (4.2)	
ZDV, ddI, and d4T use	19 (11)	14 (11)	5 (10)	0.92
HIV RNA <400 copies/mL, *n* (%)	146 (83)	105 (83)	41 (85)	0.66
Median nadir CD4 count (IQR), cells/*μ*L	168 (42–322)	156 (38–304)	197 (68–354)	0.25
Median current CD4 count (IQR), cells/*μ*L	452 (294–640)	438 (280–630)	498 (406–690)	0.09
Median BMI (IQR), kg/m^2^	25 (22–31)	24 (22–26)	36 (32–39)	<0.001
Receipt of HD vaccine, *n* (%)	91 (52)	64 (50)	27 (56)	0.46

ART: antiretroviral therapy; BMI: body mass index; ddI: didanosine; d4T: stavudine; HD: high dose; IQR: interquartile range; INSTI: integrase strand transfer inhibitor; NNRTI: nonnucleoside reverse transcriptase inhibitor; PI: protease inhibitor; ZDV: zidovudine.

**(a):** 

	Nonobese (BMI < 30 kg/m^2^)	Obese (BMI ≥ 30 kg/m^2^)	*p* value
	(*n* = 128)	(*n* = 48)	
Seroconversion, % (95% CI)			
H1N1	70 (62–78)	62 (48–77)	0.32
H3N2	77 (69–84)	79 (67–91)	0.71
Influenza B	44 (35–52)	48 (33–63)	0.62

**(b):** 

	SD vaccine recipients			HD vaccine recipients		
	Nonobese	Obese	*p* value	Nonobese	Obese	*p* value
	*n* = 64	*n* = 21		*n* = 64	*n* = 27	
Seroconversion, % (95% CI)						
H1N1	59 (47–72)	62 (39–84)	0.84	81 (71–91)	63 (43–82)	0.06
H3N2	75 (64–86)	71 (50–92)	0.74	78 (68–88)	85 (71–100)	0.44
Influenza B	34 (22–46)	29 (8–50)	0.62	53 (40–66)	63 (43–82)	0.39
Seroprotection, % (95% CI)						
H1N1	86 (77–95)	90 (77–100)	0.59	98 (95–100)	89 (76–100)	0.04
H3N2	91 (83–98)	100 (100–100)	0.15	97 (92–100)	93 (82–100)	0.36
Influenza B	83 (73–92)	67 (45–89)	0.12	89 (81–97)	96 (89–100)	0.27
Prevaccination GMT (95% CI)						
H1N1	20 (11–37)	22 (7–67)	0.90	17 (9–30)	59 (23–154)	0.02
H3N2	33 (20–55)	17 (6–48)	0.20	24 (13–43)	23 (10–54)	0.97
Influenza B	16 (10–26)	18 (8–38)	0.84	18 (11–30)	20 (11–37)	0.86
Postvaccination GMT (95% CI)						
H1N1	307 (180–524)	386 (178–839)	0.66	684 (489–955)	746 (356–1570)	0.82
H3N2	322 (202–515)	338 (192–667)	0.92	681 (439–1057)	771 (409–1456)	0.75
Influenza B	63 (42–93)	60 (25–144)	0.92	149 (109–204)	122 (78–192)	0.48

BMI: body mass index; CI: confidence interval; GMT: geometric mean titer; HD: high dose; SD: standard dose.

**Table 3:** Proportions with seroconversion and seroprotection after vaccination among participants with CD4 cell counts > 200 cells/*μ*L (*n* = 157) and CD4 cell counts > 500 cells/*μ*L (*n* = 78).

Participants with CD4 > 200 cells/*μ*L (*n* = 78)	Nonobese (BMI < 30 kg/m^2^) (*n* = 110)	Obese (BMI ≥ 30 kg/m^2^) (*n* = 47)	*p* value
Seroconversion, % (95% CI)			
H1N1	70 (61–79)	62 (47–76)	0.31
H3N2	79 (71–87)	81 (69–92)	0.80
Influenza B	45 (36–55)	49 (34–64)	0.69
Seroprotection, % (95% CI)			
H1N1	94 (89–98)	89 (80–98)	0.36
H3N2	98 (96–100)	98 (94–100)	0.90
Influenza B	92 (87–97)	85 (74–96)	0.20

Participants with CD4 > 500 cells/*μ*L (*n* = 78)	Nonobese (BMI < 30 kg/m^2^) (*n* = 54)	Obese (BMI ≥ 30 kg/m^2^) (*n* = 24)	*p* value

Seroconversion, % (95% CI)			
H1N1	68 (56–81)	75 (56–94)	0.56
H3N2	72 (60–84)	79 (62–97)	0.52
Influenza B	35 (22–48)	38 (17–58)	0.84
Seroprotection, % (95% CI)			
H1N1	93 (85–100)	96 (87–100)	0.59
H3N2	98 (94–100)	100 (100–100)	0.50
Influenza B	94 (88–100)	92 (80–100)	0.64

BMI: body mass index; CI: confidence interval.

## References

[1] http://www.who.int/mediacentre/factsheets/fs211/en/.

[2] Kunisaki K. M., Janoff E. N. (2009). Influenza in immunosuppressed populations: a review of infection frequency, morbidity, mortality, and vaccine responses. *The Lancet Infectious Diseases*.

[3] Lin J. C., Nichol K. L. (2001). Excess mortality due to pneumonia or influenza during influenza seasons among persons with acquired immunodeficiency syndrome. *Archives of Internal Medicine*.

[4] Fabbiani M., Sidella L., Corbi M. (2013). HIV-infected patients show impaired cellular immune response to influenza vaccination compared to healthy subjects. *Vaccine*.

[5] Kelly D., Burt K., Missaghi B. (2012). Responses to pandemic ASO3-adjuvanted A/California/07/09 H1N1 influenza vaccine in human immunodeficiency virus-infected individuals. *BMC Immunology*.

[6] Tebas P., Frank I., Lewis M. (2010). Poor immunogenicity of the H1N1 2009 vaccine in well controlled HIV-infected individuals. *AIDS*.

[7] Roome A. J., Walsh S. J., Cartter M. L., Hadler J. L. (1993). Hepatitis B vaccine responsiveness in Connecticut public safety personnel. *The Journal of the American Medical Association*.

[8] Young K. M., Gray C. M., Bekker L.-G. (2013). Is obesity a risk factor for vaccine non-responsiveness?. *PLoS ONE*.

[9] Weber D. J., Rutala W. A., Samsa G. P., Santimaw J. E., Lemon S. M. (1985). Obesity as a predictor of poor antibody response to hepatitis B plasma vaccine. *Journal of the American Medical Association*.

[10] Eliakim A., Schwindt C., Zaldivar F., Casali P., Cooper D. M. (2006). Reduced tetanus antibody titers in overweight children. *Autoimmunity*.

[11] Hopkins K. L., Laher F., Otwombe K. (2014). Predictors of HVTN 503 MRK-AD5 HIV-1 gag/pol/nef vaccine induced immune responses. *PLoS ONE*.

[12] Bandaru P., Rajkumar H., Nappanveettil G. (2011). Altered or impaired immune response upon vaccination in WNIN/Ob rats. *Vaccine*.

[13] Karlsson E. A., Beck M. A. (2010). The burden of obesity on infectious disease. *Experimental Biology and Medicine*.

[14] Milner J. J., Beck M. A. (2012). The impact of obesity on the immune response to infection. *The Proceedings of the Nutrition Society*.

[15] Zuckerman J. N. (2000). The importance of injecting vaccines into muscle. Different patients need different needle sizes. *The British Medical Journal*.

[16] Almond M. H., Edwards M. R., Barclay W. S., Johnston S. L. (2013). Obesity and susceptibility to severe outcomes following respiratory viral infection. *Thorax*.

[17] Cantürk Z., Cantürk N. Z., Çetinarslan B., Utkan N. Z., Tarkun I. (2003). Nosocomial infections and obesity in surgical patients. *Obesity Research*.

[18] Kwong J. C., Campitelli M. A., Rosella L. C. (2011). Obesity and respiratory hospitalizations during influenza seasons in Ontario, Canada: a cohort study. *Clinical Infectious Diseases*.

[19] van Kerkhove M. D., Vandemaele K. A. H., Shinde V. (2011). Risk factors for severe outcomes following 2009 influenza a (H1N1) infection: a global pooled analysis. *PLoS Medicine*.

[20] Callahan S. T., Wolff M., Hill H. R., Edwards K. M. (2014). Impact of body mass index on immunogenicity of pandemic H1N1 vaccine in children and adults.. *The Journal of Infectious Diseases*.

[21] Potter J. M., O'Donnell B., Carman W. F., Roberts M. A., Stott D. J. (1999). Serological response to influenza vaccination and nutritional and functional status of patients in geriatric medical long-term care. *Age and Ageing*.

[22] Sheridan P. A., Paich H. A., Handy J. (2012). Obesity is associated with impaired immune response to influenza vaccination in humans. *International Journal of Obesity*.

[23] Talbot H. K., Coleman L. A., Crimin K. (2012). Association between obesity and vulnerability and serologic response to influenza vaccination in older adults. *Vaccine*.

[24] Amorosa V., Synnestvedt M., Gross R. (2005). A tale of 2 epidemics: the intersection between obesity and HIV infection in Philadelphia. *Journal of Acquired Immune Deficiency Syndromes*.

[25] Crum-Cianflone N., Tejidor R., Medina S., Barahona I., Ganesan A. (2008). Obesity among patients with HIV: the latest epidemic. *AIDS Patient Care and STDs*.

[26] Malaza A., Mossong J., Bärnighausen T., Newell M.-L. (2012). Hypertension and obesity in adults living in a high HIV prevalence rural area in South Africa. *PLoS ONE*.

[27] Wand H., Ramjee G. (2013). High prevalence of obesity among women who enrolled in HIV prevention trials in KwaZulu-Natal, South Africa: healthy diet and life style messages should be integrated into HIV prevention programs. *BMC Public Health*.

[28] Bloomfield G. S., Hogan J. W., Keter A. (2011). Hypertension and obesity as cardiovascular risk factors among HIV seropositive patients in Western Kenya. *PLoS ONE*.

[29] McKittrick N., Frank I., Jacobson J. M. (2013). Improved immunogenicity with high-dose seasonal influenza vaccine in HIV-infected persons. a single-center, parallel, randomized trial. *Annals of Internal Medicine*.

[30] Lederman M. M., Funderburg N. T., Sekaly R. P., Klatt N. R., Hunt P. W. (2013). Residual immune dysregulation syndrome in treated HIV infection. *Advances in Immunology*.

[31] Taiwo B., Barcena L., Tressler R. (2013). Understanding and controlling chronic immune activation in the HIV-infected patients suppressed on combination antiretroviral therapy. *Current HIV/AIDS Reports*.

[32] Shah K., Alio A. P., Hall W. J., Luque A. E. (2012). The physiological effects of obesity in HIV-infected patients. *Journal of AIDS & Clinical Research*.

[33] Nolan D., Hammond E., James I., McKinnon E., Malla S. (2003). Contribution of nucleoside-analogue reverse transcriptase inhibitor therapy to lipoatrophy from the population to the cellular level. *Antiviral Therapy*.

[34] Tien P. C., Barrón Y., Justman J. E. (2007). Antiretroviral therapies associated with lipoatrophy in HIV-infected women. *AIDS Patient Care and STDs*.

[35] Morgan O. W., Bramley A., Fowlkes A. (2010). Morbid obesity as a risk factor for hospitalization and death due to 2009 pandemic influenza A(H1N1) disease. *PLoS ONE*.

[36] Lakey W., Yang L.-Y., Yancy W., Chow S.-C., Hicks C. (2013). Short communication: from wasting to obesity: initial antiretroviral therapy and weight gain in HIV-infected persons. *AIDS Research and Human Retroviruses*.

[37] Tate T., Willig A. L., Willig J. H. (2012). HIV infection and obesity: where did all the wasting go?. *Antiviral Therapy*.

